# From Croatian Roma to 1000 Genomes: The Story of the *CYP2D6* Gene Promoter and Enhancer SNPs

**DOI:** 10.3390/jpm12081353

**Published:** 2022-08-22

**Authors:** Anita Stojanović Marković, Željka Celinšćak, Maja Šetinc, Tatjana Škarić-Jurić, Marijana Peričić Salihović, Matea Zajc Petranović

**Affiliations:** Institute for Anthropological Research, 10000 Zagreb, Croatia

**Keywords:** *CYP2D6* gene, population genetics, Roma population, promoter, enhancer, regulation of transcription, pharmacogenomics, personalized medicine

## Abstract

The *CYP2D6* gene encodes an enzyme responsible for the metabolism of ~20% of clinically prescribed drugs. In this study, 18 SNPs from the enhancer and promoter regions of *CYP2D6* in 323 Roma from Croatia were genotyped, to find out whether the demographic history of Roma affected the distribution of the studied SNPs and their linkage disequilibrium (LD) values, with the major SNPs defining the *CYP2D6* star alleles. No differences were found between the three Roma groups in allele and genotype frequencies. The distribution of LD values of Roma was compared with LD values of European and Asian populations. Regulatory *CYP2D6* SNPs (rs5758550, rs28624811, rs1080985 and rs1080983) showed similar distribution and the highest LDs with rs16947 from the gene-coding region in all populations. In the promoter region, a complete LD between rs1080989 and rs28588594, and between rs1080983 and rs28624811, was found in Croatian Roma and investigated populations from 1000 genomes. A high LD was also found between rs1080985 from the promoter and rs5758550 from the enhancer region. SNP rs28735595 from the gene promoter region had the highest LD, with two gene region SNPs, rs1058164 and rs1135840. To conclude, the Croatian Roma population shows an LD pattern of the *CYP2D6* gene region similar to the 1000 Genomes European and Asian populations.

## 1. Introduction

The *CYP2D6* gene encodes a homonymous drug-metabolizing cytochrome P450 enzyme, responsible for eliminating more than 21% of clinically used drugs [1]. This gene, located on chromosome 22q13.1, is highly polymorphic and its genetic variations greatly contribute to the inter-individual variability of CYP2D6 enzyme activity, which is divided into four categories: poor metabolizer, intermediate metabolizer, normal metabolizer and ultrarapid metabolizer [2,3,4,5]. The Clinical Pharmacogenetics Implementation Consortium (CPIC) offers guidelines for assigning activity scores of *CYP2D6* variant alleles, and subsequent translations of diplotypes into phenotypes—this method was proposed and established by Gaedigk and colleagues to standardize genotype-to-phenotype translations [6]. However, recent studies have shown that even in individuals with the same genotype, CYP2D6 enzyme activity can vary up to several times [7,8,9,10], and differential regulation of *CYP2D6* transcription may partly explain the variability in *CYP2D6*-mediated drug metabolism [11]. 

According to the GeneCards database [12], there are 87 loci in enhancer and promoter regions related to the expression of the *CYP2D6* gene, spanning from less than 1000 to almost 300,000 base pairs away from the transcription starting site (TSS). Among these, eight are active in hepatocytes (https://epd.epfl.ch/, https://www.encodeproject.org/, and https://www.ncbi.nlm.nih.gov/refseq/ (accessed on 15 March 2022)). The association of enhancer/promoter activity and variations in the *CYP2D6* gene with overall drug metabolizing is not extensively studied. So far, only a few regulatory variants of the *CYP2D6* gene have been studied. Mostly, the two completely linked single-nucleotide polymorphisms (SNPs), rs133333 (G > A) and rs5758550 (G > A), located ~116 kb downstream of the gene and identified as enhancers [13]. These SNPs are located within the 2.4 kb-long enhancer GH22J042015, the binding site for the transcription factor ZNF512 [12]. In addition to the aforementioned enhancer SNPs, the other most studied SNPs are located within the promoter/enhancer GH22J042130, which is 1.6 kb in size and 0.7 kb away from the TSS of the *CYP2D6* gene. According to the Pharmacogene Variation (PharmVar) Consortium, some of the SNPs from this region are part of haplotypes that define the *CYP2D6* star alleles [14,15,16].

Considering all cell types, GH22J042130 is the biding site of 15 transcription factors and affects the transcription of 11 genes [12]. In transcription related to drug metabolizing activity, this promoter is induced by the binding of hepatocyte nuclear factor 4 alpha, Kruppel-like factor 9 and peroxisome proliferator-activated receptor alpha, and is suppressed by nitric oxide and estrogen [11]. Since studies have shown that haplotypes containing enhancer or promoter loci allow the determination of CYP2D6 enzyme activity in vivo, their inclusion in genotyping panels could allow more accurate prediction of CYP2D6 activity [12]. SNPs from enhancers and promoter regions may be in linkage disequilibrium (LD) with star allele-defining SNPs from the *CYP2D6* gene region, which may influence the metabolizing effect [17]. LD differs among populations, especially if they are isolated, have a different ancestry from the surrounding majority population and are susceptible to genetic drift. 

An example of such a population is the Roma (Gypsy) population, a transnational minority present in many countries around the world. They originated in India and arrived in Europe around the 11th century via Central Asia (Afghanistan and Persia), the Middle East and present-day Turkey. It is estimated that the Roma population is numbering around 15 million people worldwide, of whom 12 million reside in Europe. Roma in Croatia belong to two socio-culturally and linguistically different groups: Vlax Roma, descendants of the Roma who crossed the Danube River between the 13th and 15th century and arrived in Wallachia and Transylvania (both in present-day Romania), and Moldavia, where they were forced to work in the mines for the next 500 years. During that time, they were forbidden to use their own language, so their descendants are now recognized by a specific archaic Romanian language—ljimb’d bayash. The second group is Balkan Roma, descendants of the Roma who arrived in the Balkans in the 11th century, and they speak dialects of the romani chib language. Socio-cultural characteristics of the Roma population, such as strict rules of endogamy, in addition to the founder and the bottleneck effects, have caused the genetic structure of Roma to differ compared to other populations [18,19,20], which has been shown to affect ADME genes’ variations as well [21].

The main objective of this study was to estimate the variation in enhancers and promoter regions of the *CYP2D6* gene among: (a) three socio-culturally and geographically distinct Croatian Roma groups, and (b) Croatian Roma and European and Asian 1000 Genomes populations; in particular, to find out whether the specific history of the Roma population influenced the distribution of the studied SNPs and their LDs with the main *CYP2D6* star allele-defining SNPs. The knowledge of LD between *CYP2D6* star allele-defining SNPs and SNPs in promoter regions and/or enhancers can enable prediction of *CYP2D6* activity with greater accuracy.

## 2. Materials and Methods

We analyzed 323 DNA samples, all collected during field studies of the ongoing multidisciplinary anthropological, molecular-genetic and epidemiological investigations of Roma populations in Croatia. Samples belong to members of the three socio-culturally different Roma subpopulations: the Vlax Roma, who are divided into two subpopulations according to the geographical regions of Croatia they inhabit: Baranja and Medjimurje, and the Balkan Roma from the city of Zagreb. All Roma participated in the study voluntarily, and with the help of Roma volunteers, were informed about the goals, methods and expectations of the study. The Scientific Board and the Ethics Committee of the Institute for Anthropological Research in Zagreb, Croatia, approved the study protocol.

DNA was extracted from peripheral blood using the salting-out method [22]. The genotyping of 16 SNPs in the promoter region of the *CYP2D6* gene and two enhancer SNPs on chromosome 22 was carried out using the Kompetitive Allele-Specific PCR method (KASP) in a commercial facility. The KASP genotyping assay is a form of competitive allele-specific PCR combined with a homogeneous fluorescent SNP genotyping system, which determines the alleles at a specific locus within genomic DNA [23]. Data for the *CYP2D6* star allele-defining SNPs (rs1135840, rs16947, rs28371725, rs3892097, rs1058164, rs1065852 and rs769258) in Croatian Roma were taken from a paper by Stojanović Marković et al. [24].

Allele and genotype frequencies were calculated by direct counting. Hardy–Weinberg equilibrium (HWE) was assessed using the software Arlequin 3.5 [25]. Genotype and allele frequency differences between the three Roma groups were tested using the Chi-square test. The analyses were performed using R with statistical significance set at *p* < 0.05 [26]. Linkage disequilibrium (LD) analyses in the Roma groups have been performed using the software Haploview [27]. Only r^2^ values of LD were calculated since it is considered more robust than D’ and correlates better among different population samples [28,29]. Haploview software was also used for drawing plots. Data from the 1000 Genomes database were used to compare the Croatian Roma population with European and Asian populations for the SNPs studied. The European cluster consisted of the following populations: Utah residents with Northern and Western ancestry (CEU), Finland (FIN), British in England and Scotland (GBR), Iberian population in Spain (IBS) and Toscani in Italy (TSI). The East Asian cluster consisted of Dai Chinese (CDX), Han Chinese in Beijing (CHB), South Han Chinese (CHS), Japanese in Tokyo (JPT) and Kinh in Ho Chi Minh City, Vietnam (KHV), while the South Asian cluster consisted of Bengali in Bangladesh (BEB), Gujarati Indian (GIH), Indian Telugu in the UK (ITU), Punjabi in Lahore Pakistan (PJL) and Sri Lankan Tamil in the UK (STU). LDs for European, East Asian and South Asian populations were calculated using the LD calculator implemented in the Ensembl genome browser [30]. Spearman’s correlation was used to compare LD values [26]. Spearman’s correlation results were used as input for multidimensional scaling (MDS), and plots were drawn using ggplot2 [31]. 

## 3. Results

Allele and genotype frequencies of studied polymorphic sites determined in three Croatian Roma subpopulations are shown in Table 1. Eight out of the eighteen investigated SNPs in our sample were monomorphic (rs1080993, rs34894147, rs1376235338, rs1224722684, rs1409156443, rs536645539, rs1080990 and rs58188898). All polymorphic sites except for rs13333 in the Baranja Roma subpopulation were in Hardy–Weinberg equilibrium. None of the SNPs showed significant differences in genotype or allele frequencies between the three Roma groups (Table 1).

In Table 2, the linkage disequilibrium (LD) values for the three Croatian Roma subpopulations (r^2^ values) are shown for pairs of two enhancer (rs133333 and rs5758550) and six polymorphic promoter SNPs (rs28624811, rs1080989, rs28735595, rs28588594, rs1080985 and rs1080983), as well as between pairs of the latter and SNPs that define different *CYP2D6* gene star alleles (rs1135840, rs16947, rs28371725, rs3892097, rs1058164, rs1065852 and rs769258). Two SNPs in the *CYP2D6* gene promoter region, rs35046171 and rs34167214, were not included in the LD calculation due to the extremely low prevalence of minor alleles in these SNPs. 

**Figure 1:**
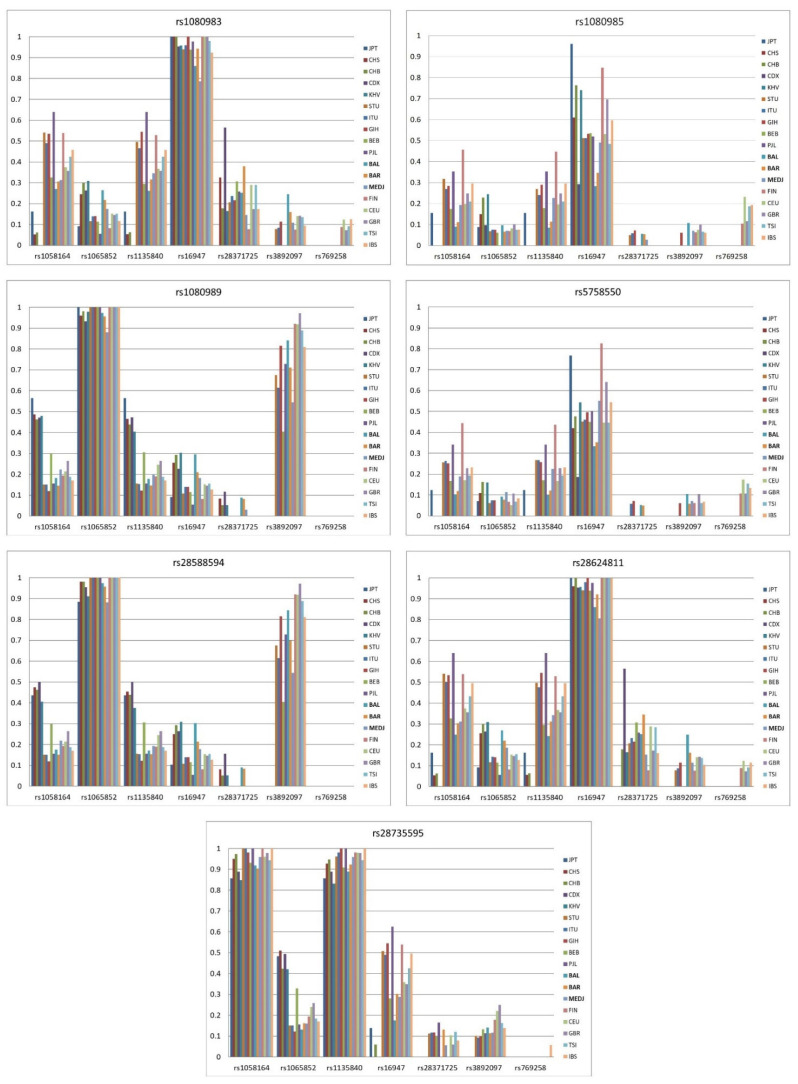
LD values’ (r^2^) distribution between the *CYP2D6* promoter and enhancer SNPs and star allele-defining SNPs in the European, South Asian, East Asian and Croatian Roma populations.

Figure 1 graphically shows the distribution of LD values between the *CYP2D6* promoter region/enhancers’ SNPs and star allele-defining SNPs in the European, South Asian, East Asian and Croatian Roma populations. 

In the studied world populations, four SNPs in the *CYP2D6* regulatory regions (rs5758550 in the enhancer region, and rs28624811, rs1080985 and rs1080983 in the promoter region) showed similar distributions and the highest LD with rs16947 from the *CYP2D6* gene region (Figure 1). Since these four regulatory region SNPs have the same LD pattern with the SNPs in the gene region, we calculated their pairwise LDs and found that promoter regions rs1080983 and rs28624811 are in complete LD not only in the European and Asian populations (data not shown), but also in the Croatian Roma population (Table 2). A high LD (r^2^ > 0.8) was found between rs1080985, from the promoter region, and rs5758550, from the enhancer region, both in world populations and Croatian Roma groups (Figure 1). Other SNP pairs have LD values ranging from 0.4 to 0.8, with the highest values in the Finnish population. Promoter region SNPs rs1080989 and rs28588594 also showed nearly identical distribution of LD values in the studied populations, and the highest LDs with rs1065852 from the *CYP2D6* gene-coding region. We tested LD values between rs1080989 and rs28588594 from the promoter region and found that they were in complete LD in all studied populations. SNP rs28735595, from the *CYP2D6* gene promoter region, had the highest LD with two SNPs in the gene region, rs1058164 and rs1135840 (r^2^ > 0.8 for both SNPs). A more precise insight into LD values in Croatian Roma groups is shown in the Supplementary Figures S1–S3, which show nine LD plots for each of the Croatian Roma subpopulations. 

**Figure 2:**
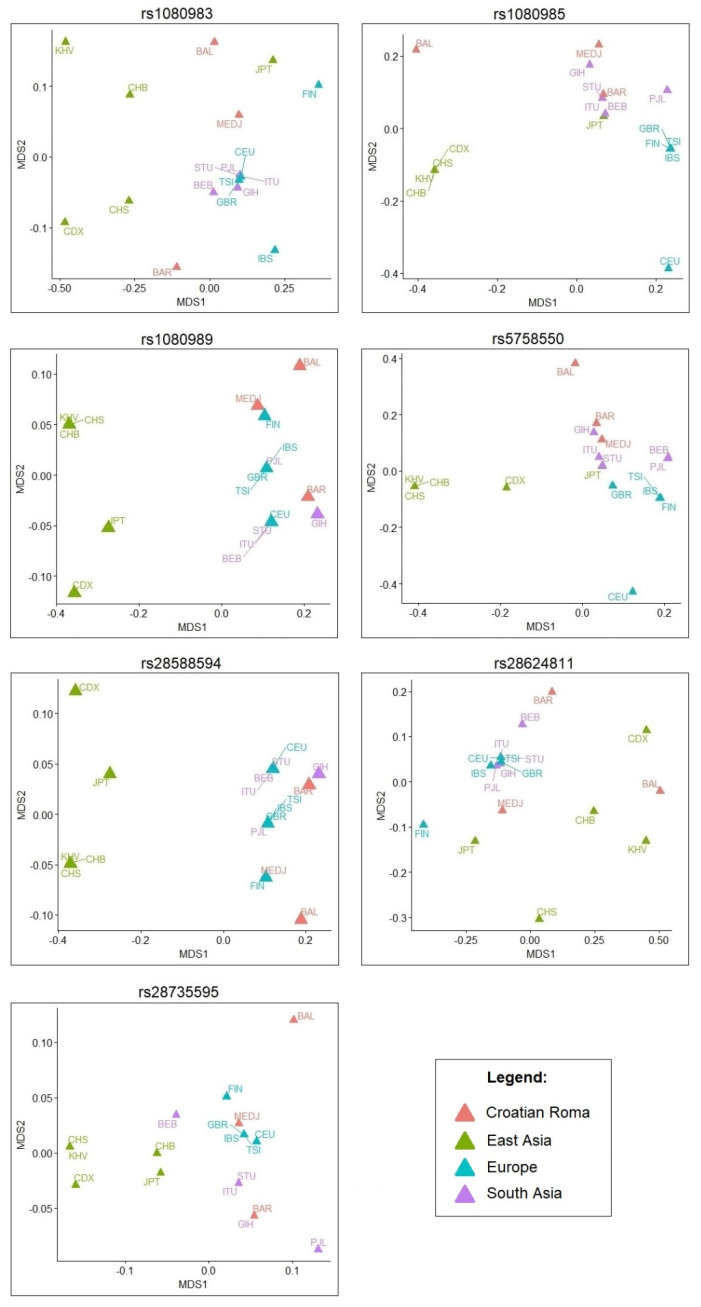
Multidimensional scaling plots (MDS) of Spearman’s correlation matrices for linkage disequilibrium (LD) between the *CYP2D6* promoter and enhancer SNPs and star allele-defining SNPs in the European, South Asian, East Asian and Croatian Roma populations.

To reveal the pattern of LD correlations, the MDS plots (Figure 2) were constructed as described in the Materials and Methods Section. Most MDS plots show separation of East Asian populations from other populations. Considering the Croatian Roma population, the plots also suggest a slightly remote position of the Balkan Roma subpopulation from others, while the Baranja Roma subpopulation is almost always positioned close to some of the South Asian populations. The Roma subpopulation from Medjimurje is positioned either close to South Asian populations (MDS plots for rs5758550, rs28624811 and rs1080985), or closer to European populations (MDS plots for rs28735595, rs28588594 and rs1080989). 

## 4. Discussion

Population pharmacogenomics is a growing area driven by increasing population data on genes responsible for absorption, distribution, metabolism and excretion (ADME genes). Population ancestry may affect the diversity of genetic polymorphisms, leading to population-specific differences in drug responses [32]. Within population pharmacogenomics, special attention should be given to the study of indigenous and/or minority populations which, due to their genetic history, show a specific distribution of alleles that can alter drug metabolism and lead to adverse drug reactions (ADR).

The pharmacogenomics of the Roma minority population has been studied for the last few years [33]. These studies included SNPs in several ADME genes, such as *ABCB1* [34,35], *CYP2B6* [36,37,38], *CYP2C19* [39,40,41,42], *CYP2D6* [24,37,38,41] and *NAT* [42,43].

Previous analyses showed that the three socio-culturally different Croatian Roma groups show significant differences in allele distribution within the *CYP2D6* gene [24], and therefore we continued to investigate promoter and enhancer SNPs associated with this gene. In general, diversity in regulatory elements has an impact on gene expression, so understanding it could help to elucidate the unexplained variability in gene activity [11]. Contrary to the differences found among Croatian Roma groups in the *CYP2D6* gene region, the regulatory elements studied here showed no difference among the same Roma subpopulations.

To clarify the relationships of SNPs in the promoter/enhancer region with star allele-defining SNPs from the *CYP2D6* gene region in the Croatian Roma population, we determined their LDs. Significant LDs between SNPs in regulatory and gene regions may affect *CYP2D6* transcription and consequently drug metabolism, and so far, the most studied example of this interaction is rs5758550 [44]. Using the reporter gene assay, Wang et al. [45] found that the constructs containing minor allele G had higher activity independently of other SNPs which were part of the construct (rs133333 and rs4822082), while deletion of the region surrounding rs5758550 decreased *CYP2D6* mRNA levels. Rs133333 and rs5758550 are in complete LD, but chromatin immunoprecipitation with the P300 antibody showed that deletion of 156 bp surrounding rs133333 did not decrease the level of transcription of *CYP2D6* [45]. SNPs rs5758550 and rs133333, genotyped in Croatian Roma subpopulations, were also in high LD. The LDs of enhancers rs5758550 and rs133333 with SNPs from the *CYP2D6* promoter region were also calculated. Only rs1080985 was in LD with the two enhancer SNPs. Raimundo et al. [46] and Zanger et al. [47] linked rs1080985 with increased *CYP2D6* expression in the human liver, but this was not supported by reporter gene assays [13]. Today, it is considered that this SNP has no functional consequences (https://www.ncbi.nlm.nih.gov/clinvar/ (accessed on 15 March 2022)). Wang et al. [13] suggested that higher levels of *CYP2D6* mRNA expression, previously thought to be associated with this SNP, may be explained by LD between rs5758550/rs133333 enhancer SNPs and rs1080985. Haplotypes reconstructed in the studied Croatian Roma population have the rs5758550 allele G and the rs1080985 allele C on more than 20% of chromosomes.

Furthermore, we investigated LDs between enhancer/promotor loci and major star allele-defining SNPs. An r^2^ LD value higher than 0.8, indicating a significant association, was found between rs1080983, which is part of the CTCF binding site, and SNP rs16947, which defines allele *2. This SNP is also in high LD with rs28624811 from the promoter/enhancer GH22J042130. SNPs from the same regulatory element, rs1080989 and rs28588594, are in high LD with allele *4 (rs3892097), but an LD value higher than 0.8 was found only in Roma from Balkan, while this high LD value has been observed in all Croatian Roma groups for allele *10 (rs1065852). CYP2D6*10 is a decreased-function allele predominantly found in East and South Asian populations, where its prevalence ranges from 9% to 44%. Its frequency in African populations is between 4% and 6%, among Europeans, <2%, and in the Croatian Roma, 6% [24,48,49]. The non-function CYP2D6*4 allele, which is predominantly found in European populations (18%), had the highest frequency in the Balkan and Baranja Roma groups, even higher than in European populations. The prevalence of the allele *4 in the Medjimurje Roma group is lower than in the European population, but still higher than in other world populations [24,48,49]. Since both these star alleles have an impaired function, an additional analysis such as the reporter gene assay could help to untangle the effect of these promoter SNPs on *CYP2D6* functionality. A high LD was also noticed between the promoter region SNP rs28735595, and SNPs rs1058164 and rs1135840, which are present in all the main *CYP2D6* star alleles. Since the Roma population has a specific genetic history, we were interested to find out whether their *CYP2D6* LD distribution is similar to other world populations. Combinations of the *CYP2D6* regulatory and gene region SNPs with high LD values in the Croatian Roma are also present in the majority of world populations, but fine differences can be noticed among Roma groups. This is especially evident for rs3892097, which defines the CYP2D6*4 allele, when in LD with rs1080989 and rs28588594 from the promoter region, and this LD is the lowest in the Medjimurje Roma group compared to the other two groups. The LD correlation matrices presented in the MDS plots mostly distinguish the populations of East Asia from other studied populations. Such separation is evident in many studies related to SNPs of the ADME genes [21,50]. Population-specific differences based on r^2^ LD values were also found by Ahsan et al. [28], but between drug response-related SNPs. 

## 5. Conclusions

Although the studied Croatian Roma groups showed significant variability of the *CYP2D6* gene variants determined so far, the prevalence of alleles in SNPs from regulatory regions did not differ between these same groups. However, linkage disequilibrium values between these regulatory regions’ loci and the *CYP2D6* gene region loci differed between the Croatian Roma groups, and the population of Medjimurje showed the lowest LD values. Higher LD values between the studied SNPs of the promoter region and the SNPs defining impaired-function star alleles *2 and *4 of the *CYP2D6* gene could be used in Roma to improve genotyping efficiency if further studies demonstrate that these promoter SNPs affect the functionality of the CYP2D6 enzyme. An overall comparison of the analyzed LD values revealed that while there was greater variety in the populations of East Asia, they were uniform in populations of Europe and South Asia and distinct in their distribution. In the future, our goal is to sequence the promoter region of the *CYP2D6* gene in Croatian Roma samples, as this would help to further elucidate the structure and frequencies of common overlapping haplotypes of the *CYP2D6* gene, as well as those specific to the Roma population.

## Figures and Tables

**Table 1:** Genotype and allele frequencies of 16 *CYP2D6* promoter and 2 enhancer SNPs in the three Croatian Roma samples (Baranja, Medjimurje and Balkan).

Polymorphisms		Genotypes and Alleles	Baranja	Medjimurje	Balkan	Total	Chi Square	*p*	HWE Baranja	HWE Medjimurje	HWE Balkan
rs133333	genotypes	A/A	78	53	56	187	8.298	0.081	0.038	0.739	0.303
		A/G	24	39	30	93					
		G/G	6	6	7	19					
	alleles	A	180	145	142	467	5.738	0.057			
		G	36	51	44	131					
rs5758550	genotypes	A/A	78	54	56	188	8.186	0.085	0.092	0.499	0.491
		A/G	27	43	33	103					
		G/G	6	6	7	19					
	alleles	A	183	151	145	479	5.549	0.062			
		G	39	55	47	141					
rs1080993	genotypes	C/C	113	107	93	313					
	alleles	C	226	214	186	626					
rs34894147	genotypes	CC/CC	112	104	96	312					
	alleles	CC	224	208	192	624					
rs1376235338	genotypes	C/C	114	104	96	314					
	alleles	C	228	208	192	628					
rs35046171	genotypes	G/G	113	104	95	312	1.019	0.601	0.963		0.959
		A/G	1	0	1	2					
	alleles	G	227	208	191	626	1.016	0.602			
		A	1	0	1	2					
rs1224722684	genotypes	G/G	114	105	96	315					
	alleles	G	228	210	192	630					
rs34167214	genotypes	A/A	113	105	94	312	0.864	0.649	0.963	0.961	
		C/A	1	1	0	2					
	alleles	A	227	211	188	626	0.861	0.650			
		C	1	1	0	2					
rs1409156443	genotypes	C/C	113	106	96	315					
	alleles	C	226	212	192	630					
rs28624811	genotypes	G/G	45	40	34	119	3.573	0.467	0.123	0.598	0.251
		G/A	44	51	41	136					
		A/A	20	13	20	53					
	alleles	G	134	131	109	374	1.392	0.499			
		A	84	77	81	242					
rs536645539	genotypes	TC/TC	112	106	93	311					
	alleles	TC	224	212	186	622					
rs1080990	genotypes	C/C	114	104	94	312					
	alleles	C	228	208	188	624					
rs1080989	genotypes	C/C	60	58	47	165	1.335	0.855	0.912	0.181	0.515
		C/T	45	35	35	115					
		T/T	8	10	9	27					
	alleles	C	165	151	129	445	0.335	0.846			
		T	61	55	53	169					
rs28735595	genotypes	C/C	46	39	45	130	3.900	0.420	0.981	0.517	0.913
		C/T	51	45	39	135					
		T/T	14	17	8	39					
	alleles	C	143	123	129	395	3.642	0.162			
		T	79	79	55	213					
rs28588594	genotypes	G/G	60	60	49	169	1.074	0.898	0.890	0.158	0.461
		G/A	45	35	35	115					
		A/A	9	10	9	28					
	alleles	G	165	155	133	453	0.273	0.873			
		A	63	55	53	171					
rs1080985	genotypes	G/G	75	55	53	183	5.757	0.218	0.085	0.523	0.697
		C/G	29	43	32	104					
		C/C	7	6	6	19					
	alleles	G	179	153	138	470	3.153	0.207			
		C	43	55	44	142					
rs58188898	genotypes	G/G	111	106	96	313					
	alleles	G	222	212	192	626					
rs1080983	genotypes	C/C	49	42	35	126	3.769	0.438	0.096	0.619	0.277
		T/C	45	50	41	136					
		T/T	20	12	19	51					
	alleles	C	143	134	111	388	1.601	0.449			
		T	85	74	79	238					

The two *CYP2D6* gene enhancer SNPs are highlighted in grey, while the other SNPs are from the promoter region. Significant Chi-square and HWE *p*-values are shown in bold.

**Table 2:** LD values (r*^2^*) between pairs of polymorphic sites in the *CYP2D6* gene regulatory and gene-coding regions in the three Croatian Roma groups (Baranja, Medjimurje and Balkan).

		Baranja	Medjimurje	Balkan
L1	L2	r^2^	r^2^	r^2^
rs133333	rs5758550	**1**	**1**	**1**
rs133333	rs1135840	0.116	0.210	0.094
rs133333	rs28371725	0.046	0.030	0.051
rs133333	rs16947	0.350	0.527	0.322
rs133333	rs3892097	0.056	0.071	0.104
rs133333	rs1058164	0.113	0.175	0.098
rs133333	rs1065852	0.075	0.114	0.089
rs133333	rs769258	0.047		0.039
rs133333	rs28624811	0.344	0.618	0.437
rs133333	rs1080989	0.077	0.131	0.119
rs133333	rs28735595	0.111	0.218	0.123
rs133333	rs28588594	0.079	0.131	0.123
rs133333	rs1080985	**0.877**	**0.920**	**0.937**
rs133333	rs1080983	0.347	0.642	0.430
rs5758550	rs1135840	0.121	0.224	0.100
rs5758550	rs28371725	0.050	0.029	0.052
rs5758550	rs16947	0.353	0.551	0.333
rs5758550	rs3892097	0.057	0.072	0.105
rs5758550	rs1058164	0.118	0.188	0.103
rs5758550	rs1065852	0.077	0.114	0.092
rs5758550	rs769258	0.043		0.036
rs5758550	rs28624811	0.353	0.635	0.442
rs5758550	rs1080989	0.079	0.130	0.119
rs5758550	rs28735595	0.115	0.232	0.130
rs5758550	rs28588594	0.081	0.130	0.123
rs5758550	rs1080985	**0.884**	**0.925**	**0.941**
rs5758550	rs1080983	0.350	0.659	0.436
rs1135840	rs28624811	0.312	0.342	0.241
rs1135840	rs1080989	0.147	0.197	0.178
rs1135840	rs28735595	**0.922**	**0.958**	**0.890**
rs1135840	rs28588594	0.153	0.194	0.171
rs1135840	rs1080985	0.114	0.227	0.086
rs1135840	rs1080983	0.315	0.345	0.262
rs28371725	rs28624811	0.345	0.153	0.251
rs28371725	rs1080989	0.082	0.031	0.089
rs28371725	rs28735595	0.132	0.056	0.043
rs28371725	rs28588594	0.084	0.030	0.090
rs28371725	rs1080985	0.054	0.028	0.056
rs28371725	rs1080983	0.379	0.146	0.252
rs16947	rs28624811	**0.921**	**0.807**	**0.861**
rs16947	rs1080989	0.210	0.183	0.296
rs16947	rs28735595	0.303	0.289	0.175
rs16947	rs28588594	0.214	0.178	0.303
rs16947	rs1080985	0.346	0.491	0.283
rs16947	rs1080983	**0.943**	0.787	**0.860**
rs3892097	rs28624811	0.161	0.115	0.249
rs3892097	rs1080989	0.710	0.543	**0.842**
rs3892097	rs28735595	0.114	0.116	0.141
rs3892097	rs28588594	0.698	0.544	**0.844**
rs3892097	rs1080985	0.043	0.070	0.107
rs3892097	rs1080983	0.159	0.108	0.245
rs1058164	rs28624811	0.303	0.312	0.248
rs1058164	rs1080989	0.145	0.222	0.183
rs1058164	rs28735595	**0.903**	**0.959**	**0.918**
rs1058164	rs28588594	0.151	0.218	0.176
rs1058164	rs1080985	0.111	0.192	0.090
rs1058164	rs1080983	0.306	0.312	0.270
rs1065852	rs28624811	0.219	0.186	0.269
rs1065852	rs1080989	**0.956**	**0.880**	**0.973**
rs1065852	rs28735595	0.162	0.160	0.132
rs1065852	rs28588594	**0.957**	**0.881**	**0.973**
rs1065852	rs1080985	0.066	0.070	0.096
rs1065852	rs1080983	0.217	0.175	0.264
rs769258	rs28624811	0.015		0.015
rs769258	rs1080989	0.003		0.002
rs769258	rs28735595	0.005		0.001
rs769258	rs28588594	0.003		0.001
rs769258	rs1080985	0.038		0.039
rs769258	rs1080983	0.015		0.016
rs28624811	rs1080989	0.226	0.210	0.284
rs28624811	rs28735595	0.332	0.360	0.309
rs28624811	rs28588594	0.230	0.213	0.290
rs28624811	rs1080985	0.371	0.567	0.401
rs28624811	rs1080983	**0.981**	**1**	**1**
rs1080989	rs28735595	0.167	0.190	0.181
rs1080989	rs28588594	**1**	**1**	**1**
rs1080989	rs1080985	0.068	0.095	0.127
rs1080989	rs1080983	0.223	0.206	0.282
rs28735595	rs28588594	0.172	0.190	0.170
rs28735595	rs1080985	0.134	0.231	0.096
rs28735595	rs1080983	0.336	0.360	0.307
rs28588594	rs1080985	0.071	0.091	0.127
rs28588594	rs1080983	0.227	0.200	0.285
rs1080985	rs1080983	0.376	0.582	0.404

High LD values (r^2^ > 0.8) are shown in bold.

## Data Availability

All data analyzed in this study are available at: http://roma.inantro.hr/en/. In case of using this database for further analyses, please cite this publication. If further clarification is required, contact the corresponding author.

## Supplementary Materials

The following supporting information can be downloaded at: https://www.mdpi.com/article/10.3390/jpm12081353/s1, Figure S1: LD plots for Baranja Roma population, Figure S2: LD plots for Medjimurje Roma population, Figure S3: LD plots for Balkan Roma population.
