# Supplementary figures and images for: From Croatian Roma to 1000 Genomes: The Story of the CYP2D6 Gene Promoter and Enhancer SNPs
Source: J Pers Med. 2022 Aug 22;12(8):1353. doi: 10.3390/jpm12081353 (PMC9409800; doi:10.3390/jpm12081353)

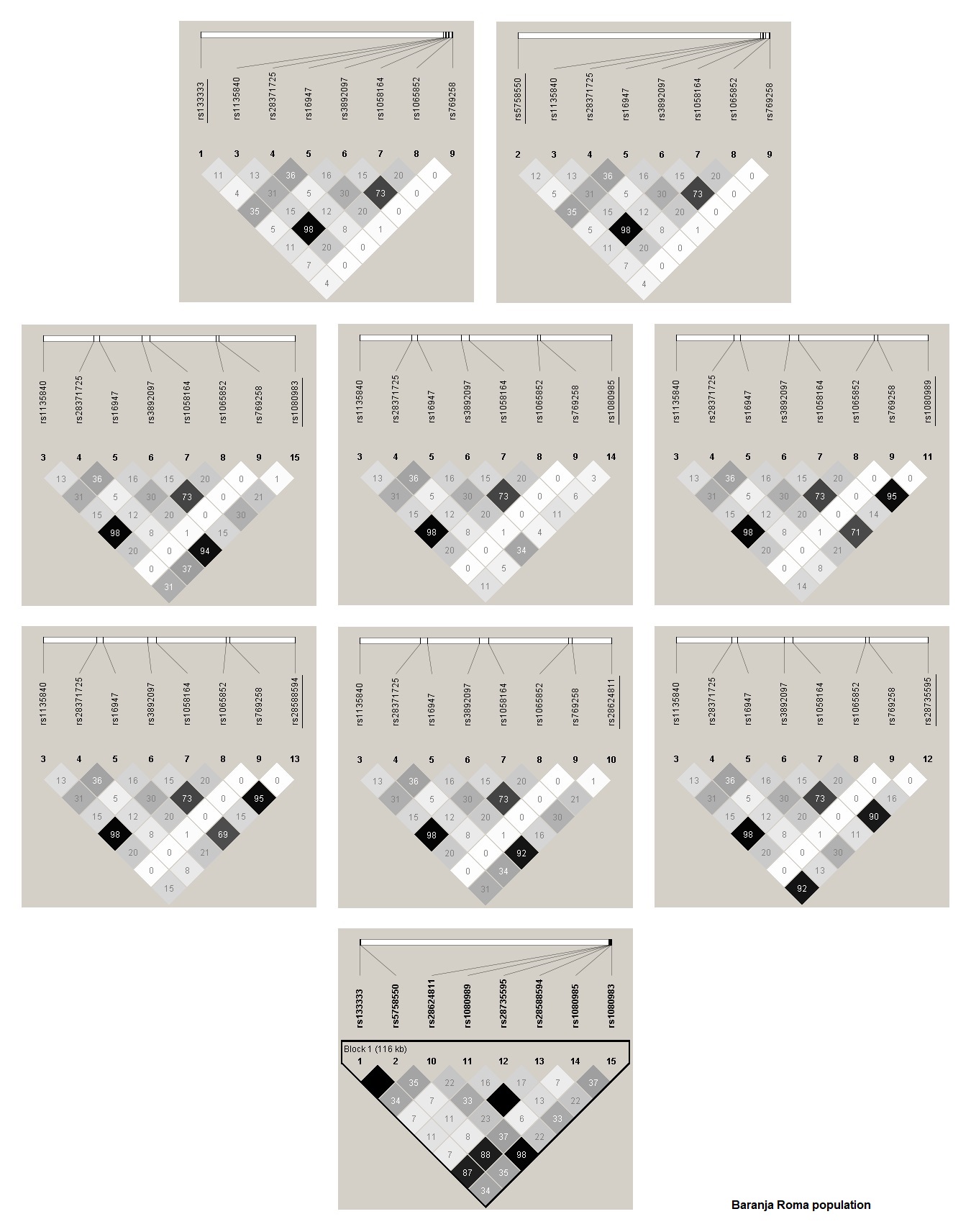

Supplement: Supplementary file 1 [file jpm-12-01353-s001.zip › Figure S1.jpg]

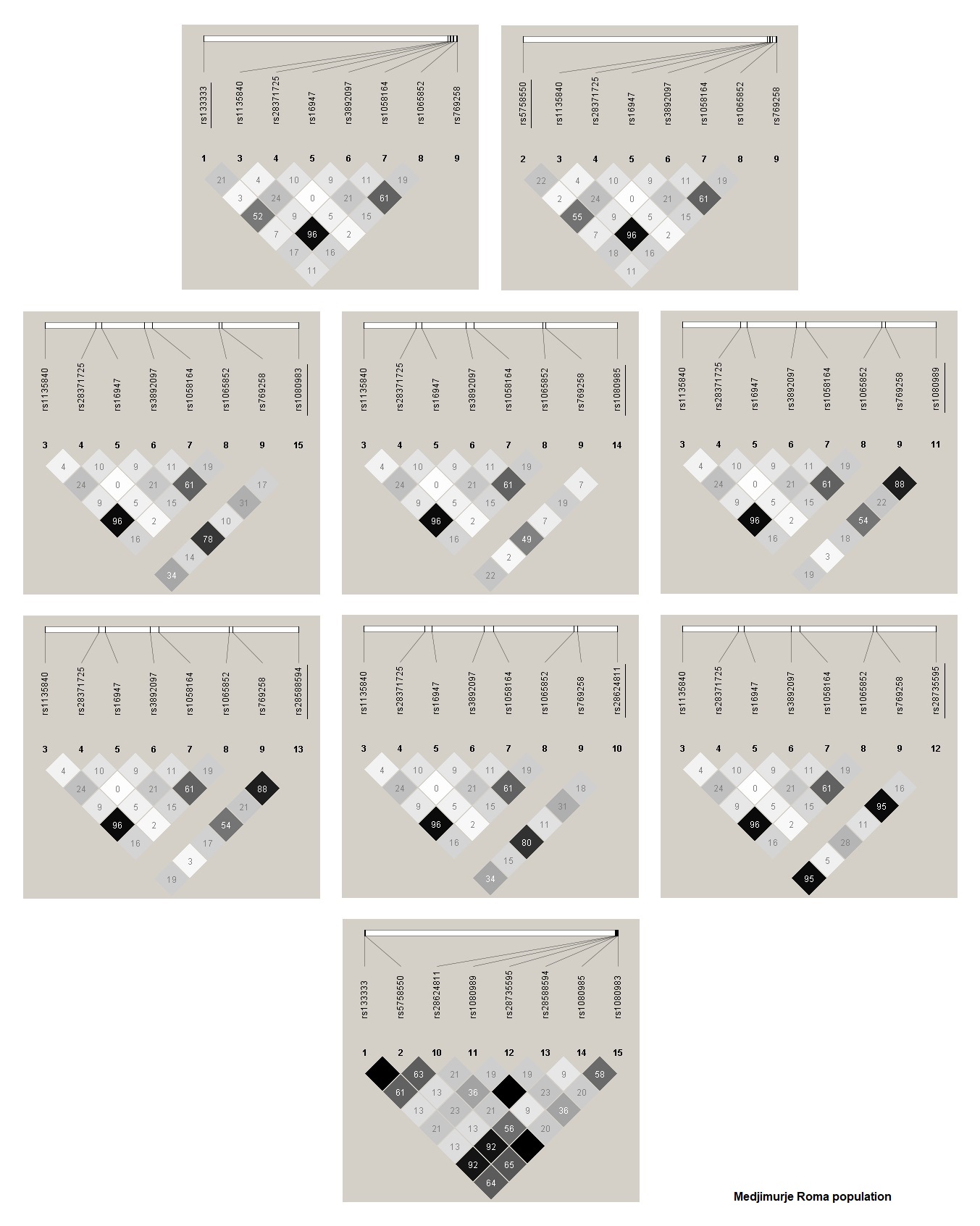

Supplement: Supplementary file 1 [file jpm-12-01353-s001.zip › Figure S2.jpg]

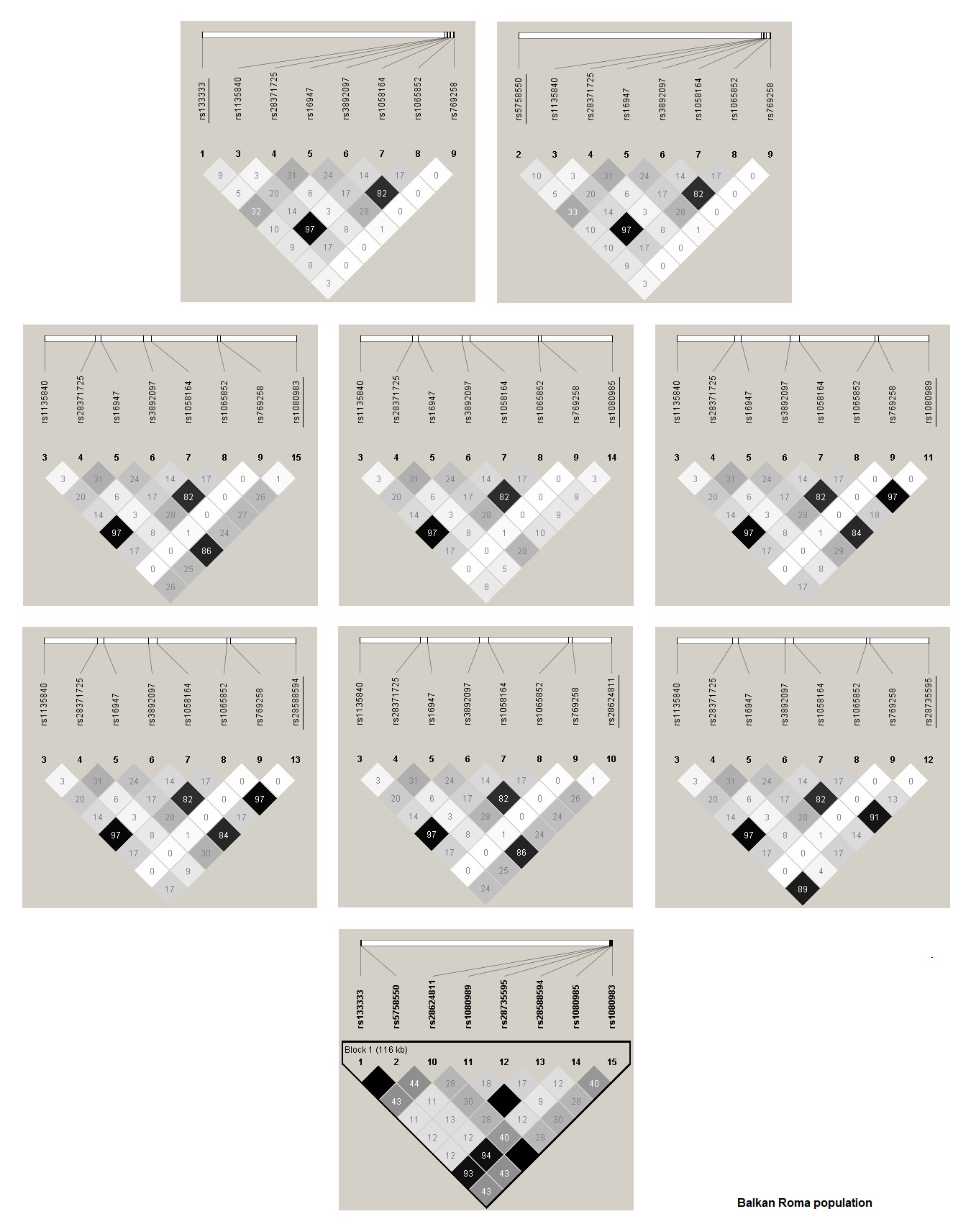

Supplement: Supplementary file 1 [file jpm-12-01353-s001.zip › Figure S3.jpg]
